# Facile synthesis of *α*-alkoxymethyltriphenylphosphonium iodides: new application of PPh_3_/I_2_

**DOI:** 10.1186/s13065-018-0421-6

**Published:** 2018-05-17

**Authors:** Humaira Yasmeen Gondal, Zain Maqsood Cheema, Javid Hussain Zaidi, Sammer Yousuf, M. Iqbal Choudhary

**Affiliations:** 1 Department of Chemistry, University of Sargodha, Sargodha, 40100 Pakistan; 2 Department of Chemistry, University of Sheffield, Sheffield, UK; 3 Department of Chemistry, Quaid-i-Azam University, Islamabad, 45320 Pakistan; 4 H.E.J Research Institute of Chemistry, ICCBS, University of Karachi, Karachi, 75270 Pakistan

**Keywords:** Bis-alkoxymethane, PPh_3_/I_2_, Quaternary phosphonium salts, O,P-acetals, Carbon homologation, Alkoxymethylphosphonium iodides

## Abstract

An efficient one pot method for the synthesis of *α*-alkoxymethylphosphonium iodides is developed by using PPh_3_/I_2_ combination at room temperature. Reaction conditions are found general to synthesize wide range of structurally variant alkoxymethylphosphonium iodides in high yield (70–91%). These new functionalized phosphonium salts are further used in stereoselective synthesis of vinyl ethers as well as in carbon homologation of aldehydes.

**Electronic supplementary material:**

The online version of this article (10.1186/s13065-018-0421-6) contains supplementary material, which is available to authorized users.

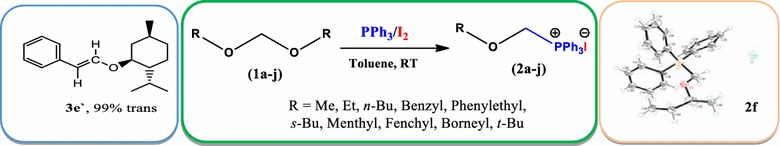

## Introduction

**Scheme 1:**
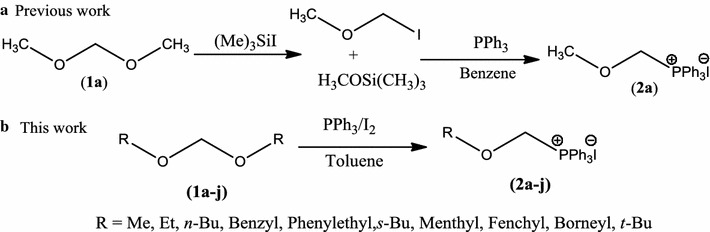
Synthesis of *α*-alkoxymethyl triphenylphosphonium iodides **2**

Functionalized phosphonium salts are gaining much attention for their diverse applications in organic synthesis [1–5]. *α*-Alkoxymethyl phosphonium salts are largely used for carbon homologation to carbonyl compounds [6–10] and also as significant synthetic intermediates [11–17]. Recently, unique reactivity of this class has been explored in nucleophilic substitution [18–20] and in novel phenyl transfer reactions [21, 22]. Methoxymethyltriphenylphosphonium chloride is commercially available salt from this class, but problem associated with its preparation involve toxic intermediate, higher temperature and long reaction time [9, 11, 23]. In perspective of alternative derivatives; α-methoxymethyl triphenylphosphonium iodide was reported by reaction of *bis*-methoxymethane (**1a**) with TMSI, followed by phosphination of methoxymethyl iodide in benzene (Scheme 1a) [24]. This only available method for iodide analogue also involves sensitive and toxic; reagent, solvent as well as intermediate along with difficult purification of product. In past few years, PPh_3_/I_2_ combination has successfully facilitated many functional groups inter-conversions [25–32]. Therefore, we decided to explore reactivity of PPh_3_/I_2_ with *bis*-alkoxymethanes (**1**) and herein efficient synthesis of a broad range of structurally diverse *α*-alkoxymethyl triphenylphosphonium iodides (**2**) is being reported (Scheme 1b). To best of our knowledge, this is the first report on general one pot synthesis of *O,P*-acetals, directly from dioxacetals on employing PPh_3_/I_2_ combination (Scheme 1b).

## Results and discussion

Current study was initiated from the model reaction of *bis*-butoxy methane (**1a**) with PPh_3_/I_2_ combination under different conditions (Table 1). Our preliminary attempt was encouraging, where 27% desired conversion (**2a**) was observed on refluxing equal molar amounts of acetal (**1a**) and PPh_3_/I_2_ in toluene for an hour (Table 1, entry 1). To improve the yield, reaction time was increased up to 3 h but only 33% required conversion was observed (Table 1, entry 2). Low yield might be associated with the sublimation of iodine at high temperature therefore, it was considered to decrease the reaction temperature. To our delight, yield was increased to 55% when the same experiment was performed at room temperature (Table 1, entry 3). Increasing the amount of PPh_3_ to 2 equivalent and reaction time up to 5 h further improved the yield (80%) (Table 1, entry 4). However, further attempts with increase in reaction time and replacing toluene with acetonitrile or solvent free conditions, were not effectual (Table 1, entry 5–8).

**Table 1:** Conditions optimization for conversion of dioxacetal to *O,P*-acetal (**2a**)

Entry	Solvent	Time (h)	Temperature (°C)	Yield (%)
1	Toluene	01	80	27
2	Toluene	03	80	33
3	Toluene	03	Room temp	55
*4* ^a^	*Toluene*	*05*	*Room temp*	*80*
5	Toluene	06	Room temp	69
6	–	02	Room temp	35
7	Acetonitrile	04	40	17
8	Acetonitrile	02	80	Traces

^a^Best optimized conditions

**Fig. 1:**
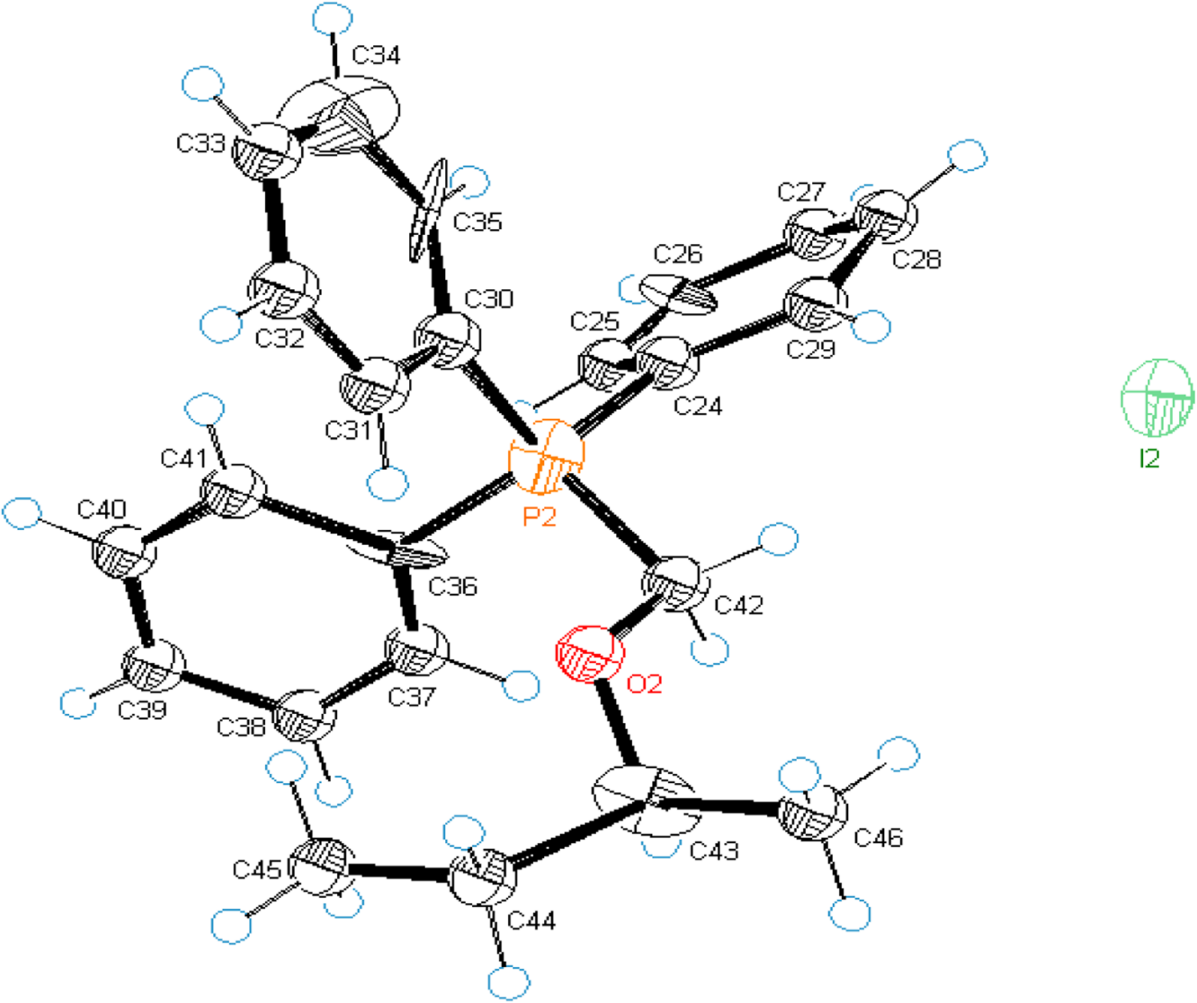
ORTEP diagram of (*S*)-2-*sec*-butoxymethyltriphenylphosphonium iodide **2f**

To explore the substrate scope of this reaction, optimized conditions were employed to structurally different *bis*-alkoxy methanes (**1a**–**j**, see Additional file 1) [33]. The method was found equally efficient to obtain broad range of alkoxymethylphosphonium iodides (**2a**–**j**, Table 2) based on primary, secondary, tertiary and benzylic alkoxy groups. Acetals having simple methoxy, ethoxy, benzoxy and phenylethoxy groups provided desired *O,P*-acetals **2b**–**e** in 75–87%. Similarly, when acetal of (*S*)-2-butanol was reacted with PPh_3_/I_2_, corresponding salt **2f** was obtained in 90% yield with retention in configuration, which was ultimately confirmed by X-ray diffraction analysis (Fig. 1).

**Table 2:**
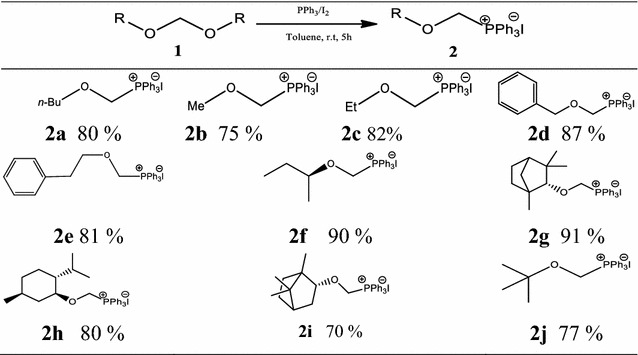
PPh_3_/I_2_ mediated synthesis of alkoxymethylphosphonium iodides (**2a**–**j**)

Optimized reaction conditions were further extended to cyclic chiral alkoxy groups including fenchyl, menthyl and borneyl, where respective chiral phosphonium salts **2g**–**i** were obtained in good yields (Table 2).

Here, (+)-menthoxymethyltriphenylphosphonium iodide **2h** is worth mentioning as its chloride analogue was prepared by tedious methodology with long reaction time [12]. Interestingly, the reaction was also successful with acetal of *t*-butanol where corresponding salt **2j** was produced in 77% yield (Table 2).

**Scheme 2:**
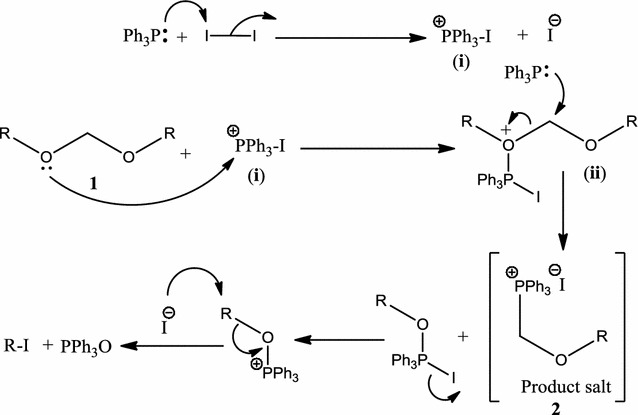
Plausible mechanism for the preparation of alkoxymethylphosphonium iodides **2**

In terms of mechanism, we envision that initially I_2_ and PPh_3_ generate phosphonium intermediate (**i**), which reacts with *bis*-alkoxymethane **1** to provide oxonium intermediate (**ii**) (Scheme 2). Another equivalent of PPh_3_ attack on oxonium intermediate (**ii**) to transform it into the target *O,P*-acetal **2** (Scheme 2).

After having a range of alkoxymethylphosphonium iodides in hand, we further explored their applications in organic synthesis. Vinyl ethers also known as enol ethers are considered important synthetic targets for the organic chemists. They itself are part of many natural products and also involve as intermediate in their total synthesis [34–36]. They act as key intermediates in many important organic reactions like Diels–Alder reaction [37], Coupling reaction [38–43], Olefin metathesis [44], Claisen rearrangement [45, 46] and Nazarov cyclization [47, 48]. They are also used in materials sciences due to their polymerization ability through cationic mechanism [49]. Despite extensive applications of enol ethers, still there is lack of general and direct method for their synthesis. Metal-catalyzed couplings are the most common available method [50–54], along with some other indirect methodologies [55–62]. Direct synthesis of enol ethers by a Wittig reaction with alkoxymethylphosphonium salt is though an evident concept but no systematic study is found in literature. Most often commercially available methoxymethylphosphonium chloride is used [63, 64], whereas effect of other alkoxy groups as well as counter anions is still need to explore. For this purpose, at first ethoxymethyltriphenylphosphanium iodide **2c** was reacted with benzaldehyde and its derivatives in the presence of *n*-BuLi, which afforded corresponding vinyl ethers **3a**–**d** (Table 3) in good yield (67–71%) and selectivity (69–73% trans).

**Table 3:**
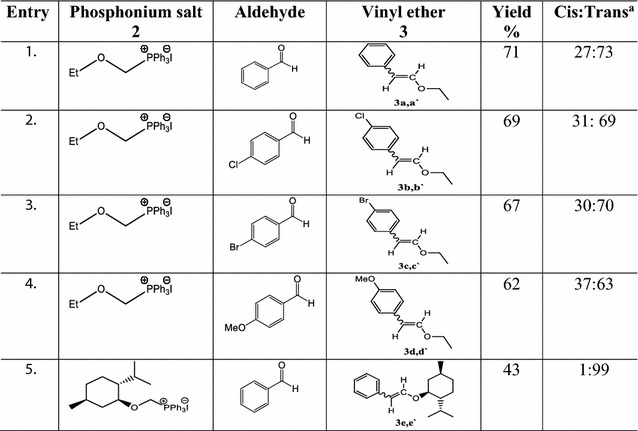
*α*-Alkoxymethylphosphonium iodides **2** in synthesis of vinyl ethers **3**

^a^Determined by ^1^H-NMR

Providentially, trans isomer **3e′** was obtained almost exclusively (99% selectivity) with (+)-menthoxymethyltriphenylphosphonium iodide **2h**. Earlier, Fuwa and Sasaki obtained same isomer **3e′** in 9% yield along with 36% cis isomer **3e** through Pd coupling [40].

Further, cost effective *n*-butoxymethylphosphonium iodide **2a** was employed for carbon homologation, where both aliphatic and aromatic aldehydes were successfully converted to higher analogous **4** in good yield (Table 4). Results show that these directly prepared and environmentally benign salts are good alternative to their chloride analogues.

**Table 4:** *α*-Butoxymethylphosphonium iodide **2a** in carbon homologation of aldehydes

Entry	Substrate	Product (**4**)	Yield (%)
1.	PhCHO	PhCH_2_CHO	72
2.	EtCHO	*n*-PrCHO	71
3.	*n*-PrCHO	*n*-BuCHO	73
4.	*n*-BuCHO	*n*-PentCHO	70

**Scheme 3:**
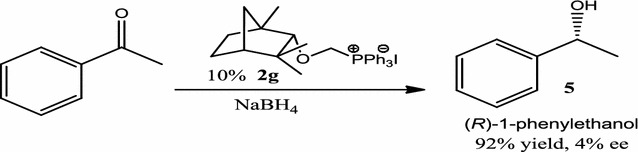
*α*-Alkoxymethylphosphonium iodide **2g** in asymmetric reduction

To evaluate catalytic potential of chiral phosphonium salts in asymmetric reduction of acetophenone, initially 10 mol% of **2g** with NaBH_4_ provided (*R*)-1-phenylethanol with 92% yield and 4% ee (Scheme 3).

Detailed study and further investigation on the application of these structurally unique *α*-alkoxymethylphosphonium salts in stereoselective synthesis of enol ethers carrying chiral auxillaries as well as in other related fields are currently underway in our laboratory.

## Conclusion

In conclusion, a facile general method for the synthesis of *α*-alkoxymethyl triphenylphosphonium iodides is developed under very mild conditions. This protocol demonstrates PPh_3_/I_2_ mediated green route to functionalized phosphonium salts. Major advantage of this methodology is to avoid toxic reagent and intermediate. These easily prepared salts were successfully employed for stereoselective synthesis of enol ethers as well as for carbon homologation in aldehydes. The new methodology will be useful for organic synthetic chemists as well as others working in associated fields.

## Experimental

All experiments were carried out under inert atmosphere using standard Schlenk technique with oven dried glassware and magnetic stirring. All solvents were freshly dried and distilled before use. All chemicals were purchased from Sigma Aldrich, Alfa Aesar and Merck. IR spectra were measured on a Perkin–Elmer Paragon 1000 (thin film) or on a Perkin–Elmer BXII spectrometer (neat). Bruker Avance NMR spectrometer of 300, 400 and 500 MHz were used for NMR spectral studies. Optical rotation was measured on Polarimeter P-2000. Crystal structure was confirmed by single crystal X-ray diffractometer Bruker Enrauf–Nonius Apex smart and Siemens P4. Mass spectra were measured on GC–MS 5977A, MAT312-EI, JEOL-600H-2, and JEOL MS-600H-1. Reactions were monitored by TLC plates from Merck (silica gel 60 F_254_, aluminum oxide 60 F_254_). TLCs were visualized by UV fluorescence and phosphomolybdic acid spraying reagent.

### General procedure for synthesis of α-alkoxymethyltriphenylphosphonium iodides (**2a**–**j**)

In a seal tube triphenylphosphine (20 mmol) and iodine (1.1 equiv) were taken in toluene (4 mL) and mixture was allowed to stir for 5 min. Solution of *bis*-alkoxymethane (**1**, 10 mmol in 1 mL toluene) was added to the reaction mixture and allowed to stir for 5 h at room temperature (28 °C). After completion of reaction, solvent was removed under reduced pressure and residue was washed with hexane to obtain required salt.

#### Butoxymethyltriphenylphosphonium iodide (**2a**)

Lemon yellow thick oil, yield = 80%, IR: υ (cm^−1^) = 689, 730, 1115, 1302, 1412, 2835. ^1^H-NMR (300 MHz, MeOD): δ ppm. 7.93–7.91 (3H, m, CH aromatic), 7.90–7.89 (3H, m, CH aromatic), 7.88–7.79 (2H, m, CH aromatic), 7.78–7.76 (3H, m, CH aromatic), 7.76–7.75 (3H, m, CH aromatic), 7.74–7.72 (1H, m, CH aromatic), 5.40 (2H, d, *J *= 4.8, CH_2_), 3.71 (2H, t, *J* = 6.4, CH_2_), 1.56–1.51 (2H, m, CH_2_), 1.28–1.22 (2H, m, CH_2_), 0.84 (3H, t, *J* = 7.6, CH_3_). ^13^C-NMR (75 MHz, MeOD): δ ppm. 136.62, 136.60 (2 carbons), 135.25, 135.15, 133.74 (3 carbons), 133.08, 132.97 (3 carbons), 131.55, 131.42, 130.01, 129.89 (2 carbons), 118.60, 117.74, 75.88, 35.76, 20.07, 13.99. ^31^P (202 MHz, CDCl_3_): δ ppm 18.83. EIMS = 349 (M^+^-I), 277.2 (48.4%), 262.2 (100%), 183.1 (59.6%), 108.0 (57.2%), 56 (36.3%).

#### Methoxymethyltriphenylphosphonium iodide (**2b**) [25]

Lemon yellow thick oil, yield = 73%, IR: υ (cm^−1^) = 691, 724, 1112, 1437, 2877, 2958. ^1^H-NMR (300 MHz, CDCl_3_): δ ppm 7.69–7.66 (3H, m, C–H aromatic), 7.65–7.61 (5H, m, C–H aromatic), 7.59–7.57 (2H, m, C–H aromatic), 7.56–7.51 (5H, m, C–H aromatic), 5.56 (2H, d, *J* = 3.9, CH_2_), 3.51 (3H, s, CH_3_). ^13^C-NMR (75 MHz, CDCl_3_): δ ppm. 135.77, 135.39, 135.35, 134.34 (3 carbons), 133.97, 133.84, 133.62, 133.49 (2 carbons), 130.78, 130.47, 130.30, 130.05, 129.89 (3 carbons), 116.85, 66.19. ^31^P (202 MHz, CDCl_3_): δ ppm 17.53. EIMS = 307 (M^+^-I), 277.2 (100%), 262.2 (67.6%), 183.1 (54.9%), 108.0 (10.9%), 77.0 (9.8%), 50.9 (5.6%).

#### Ethoxymethyltriphenylphosphanium iodide (**2c**)

Colorless oil: yield = 82%, IR: υ (cm^−1^) = 2846, 2794, 1946, 1586, 1484, 1437, 1317, 1112, 1092; ^1^H NMR (400 MHz, CDCl_3_) δ = 7.77–7.70 (9H, m), 7.65–7.60 (6H, m), 5.72 (2H, d, *J* = 3.96), 3.85 (2H, q, *J* = 7.0), 1.09 (3H, t, *J* = 7.0); ^13^C NMR (100 MHz, CDCl_3_) δ = 135.3, 135.3, 134.0 (3C), 133.9, 132.0 (3C), 131.9, 130.4, 130.3 (3C), 128.5, 128.4 (3C), 116.5, 64.21, 14.93; ^31^P-NMR (CDCl_3_): δ 25.77; HRMS +ESI calculated for C_21_H_22_OP: 321.1403; found 321.1404.

#### Benzoxymethyltriphenylphosphonium iodide (**2d**)

Yellow thick oil, yield = 87%, IR υ (cm^−1^) = 681, 734, 1103, 1305, 1425, 2767. ^1^H-NMR (300 MHz, MeOD): δ ppm. 7.82–7.76 (3H, m, CH aromatic), 7.75–7.71 (3H, m, CH aromatic), 7.70–7.67 (3H, m, CH aromatic), 7.66–7.62 (2H, m, CH aromatic), 7.58–7.55 (3H, m, CH aromatic), 7.48–7.45 (3H, m, CH aromatic), 7.37–7.29 (3H, m, CH aromatic), 5.72 (2H, d, *J *= 3, CH_2_), 4.97 (2H, s, CH_2_). ^13^C-NMR (75 MHz, MeOD): δ ppm. 134.57 (3 carbons), 133.60 (4 carbons), 133.19 (3 carbons), 132.33, 131.91 (4 carbons), 130.49 (4 carbons), 129.78, 129.41 (4 carbons), 97.76, 78.39. ^31^P (202 MHz, MeOD): δ ppm 17.55. EIMS = 383 (M^+^-I), 277.2 (59.6%), 262.2 (100%), 183.1 (48.4%), 108.0 (10.9%), 50.9 (9.8%).

#### Phenethoxymethyltriphenylphosphonium iodide (**2e**)

Yellowish powder, m.p = 171–173 °C, yield = 81%, IR υ (cm^−1^) = 690, 730, 1124, 1317, 2917. ^1^H-NMR (300 MHz, CDCl_3_): δ ppm. 7.78–7.36 (20H, m, CH aromatic), 5.45 (2H, d, *J *= 1.2 Hz, CH_2_), 4.21 (2H, t, *J* = 6.4, CH_2_), 2.75 (2H, t, *J* = 7.2, CH_2_). ^13^C-NMR (75 MHz, CDCl_3_): δ ppm. 138.43 (4 carbons), 137.98, 137.81 (2 carbons), 137.23, 136.31 (4 carbons), 136.06, 135.78, 135.23, 134.94 (3 carbons), 134.24, 129.81 (2 carbons), 129.12 (2 carbons), 117.89, 94.67, 77.78, 37.54. ^31^P (202 MHz, CDCl_3_): δ ppm 17.74. EIMS = 397 (M^+^-I), 277.2 (100%), 262.2 (67.6%), 183.1 (59.6%), 108.0 (13.4%), 91 (43%).

#### (*S*)-*sec*-Butoxymethyltriphenylphosphonium iodide (**2f**)

Yellowish white crystals, m.p = 58 °C, yield = 89%, $\left[ \alpha \right]_{D}^{25}$ = − 11 (*c* = 0.0018, MeOH), IR: υ (cm^−1^) = 682, 709, 1107, 1311, 1444, 2863. ^1^H-NMR (300 MHz, MeOD): δ ppm. 7.93–7.88 (3H, m, CH aromatic), 7.85–7.83 (1H, m, CH aromatic), 7.82–7.78 (3H, m, CH aromatic), 7.77–7.67 (3H, m, CH aromatic), 7.64–7.63 (3H, m, CH aromatic), 7.63–7.60 (1H, m, CH aromatic), 7.56–7.54 (1H, m, CH aromatic), 5.51 (1H, dd, *J *= 13.5, 4.8, CH_2_), 5.39 (1H, dd, *J* = 13.5, 5.7, CH_2_), 3.70–3.64 (1H, m, CH), 1.60–1.43 (2H, m, CH_2_), 1.18 (3H, d, *J* = 6.0 Hz, CH_3_), 0.75 (3H, t, *J* = 7.5, CH_3_). ^13^C-NMR (75 MHz, MeOD): δ ppm. 139.32, 135.11, 134.98, 134.72, 134.46, 133.95, 133.60, 133.32, 133.00, 132.93, 132.60, 131.82, 131.27, 130.87, 130.04, 129.90, 129.83, 128.60, 94.89, 79.51, 30.51, 20.10, 10.09. ^31^P (202 MHz, CDCl_3_): δ ppm 19.01. EIMS = 349 (M^+^-I), 277.2 (7.7%), 262.2 (55.9%), 183.1 (100%), 167.1 (49.8%), 152.1 (14.8%), 108.0 (13.4%), 91.0 (43.9%).

#### Triphenyl((((2R)-1,3,3-trimethylbicyclo[2.2.1]heptan-2-yl)oxy)methyl) phosphonium iodide (**2g**)

Lemon yellow thick oil, yield = 91%, $\left[ \alpha \right]_{D}^{25}$ = + 55 (*c* = 0.004, MeOH), IR: υ (cm^−1^) = 684, 968, 1112, 2948. ^1^H-NMR (300 MHz, MeOD): δ ppm. 7.89–7.83 (4H, m, CH aromatic), 7.82–7.80 (1H, m, CH aromatic), 7.80–7.63 (4H, m, CH aromatic), 7.61–7.55 (3H, m, CH aromatic), 7.54–7.51 (3H, m, CH aromatic), 5.53 (2H, dd, *J *= 1.2, 4.8 Hz, CH_2_), 3.10 (1H, d, *J* = 14.1, CH), 1.67–1.53 (2H, m, CH_2_), 1.49–1.37 (2H, m, CH_2_), 1.06–1.01 (1H, m, CH), 1.06–0.96 (2H, m, CH_2_), 0.91 (3H, s, CH_3_), 0.83 (3H, s, CH_3_), 0.73 (3H, s, CH_3_). ^13^C-NMR (75 MHz, CDCl_3_): δ ppm. 135.48, 135.45 (2 carbons), 134.26, 134.18, 132.06 (3 carbons), 132.01, 131.99, 130.53 (3 carbons), 130.43, 128.56, 128.47, 116.88, 116.20, 98.49, 66.63, 49.50, 48.38, 41.18, 40.01, 31.10, 26.10, 25.80, 20.72, 19.93. ^31^P (202 MHz, CDCl_3_): δ ppm 19.46. EIMS = 429 (M^+^-I), 277.2 (48.4%), 262.2 (100%), 183.1 (59.6%), 108.0 (57.2%), 56 (36.3%).

#### ((((1S,2R)-2-isopropyl-5-methylcyclohexyl)oxy)methyl)triphenylphosphonium iodide (**2h**)

Light yellow semisolid, yield = 80%, $\left[ \alpha \right]_{D}^{25}$ = + 8 (*c* = 0.027, MeOH), IR: υ (cm^−1^) = 687, 963, 1112, 2914. ^1^H-NMR (300 MHz, MeOD): δ ppm. 7.91–7.90 (2H, m, CH aromatic), 7.89–7.88 (1H, m, CH aromatic), 7.88–7.87 (2H, m, CH aromatic), 7.86–7.83 (4H, m, CH aromatic), 7.80–7.75 (1H, m, CH aromatic), 7.73–7.32 (1H, m, CH aromatic), 7.31–7.30 (1H, m, CH aromatic), 7.28–7.27 (1H, m, CH aromatic), 7.30–7.25 (1H, m, CH aromatic), 7.23–7.22 (1H, m, CH aromatic), 5.62 (1H, dd, *J* = 6.7, 3.3, CH_2_), 5.24 (1H, dd, *J* = 6.9, 2.9, CH_2_), 3.44 (1H, td, *J* = 5.7, 9.6, CH), 2.32–2.23 (1H, m, CH), 1.69–1.57 (2H, m, CH_2_), 1.41–1.33 (2H, m, CH_2_), 1.23–1.19 (2H, m, CH_2_), 0.95–0.91 (1H, m, CH), 0.79 (3H, d, *J* = 6.9, CH_3_), 0.75 (3H, d, *J* = 6.9, CH_3_), 0.56 (3H, d, *J* = 6.9, CH_3_). ^13^C-NMR (75 MHz, CDCl_3_): δ ppm. 135.32, 135.28, 134.32, 134.18, 134.02, 133.87, 133.61, 132.20, 132.07, 130.48, 130.32, 128.86, 128.66, 128.59, 128.50, 117.41, 116.27, 83.81, 74.16, 48.28, 46.95, 40.90, 39.42, 34.21, 31.19, 25.57, 23.41, 22.27, 16.18. ^31^P (202 MHz, MeOH): δ ppm 19.19. EIMS = 431 (M^+^-I), 277.2 (100%), 262.2 (67.6%), 183.1 (54.9%), 108.0 (10.9%), 77 (9.8%), 56 (36.3%).

#### Triphenyl((((2R)-1,7,7-trimethylbicyclo[2.2.1]heptan-2-yl)oxy)methyl) phosphonium iodide (**2i**)

Light brown semi solid, yield = 70%, $\left[ \alpha \right]_{D}^{25}$ = + 2.13 (*c* = 5 mg/15 mL MeOH), IR: υ (cm^−1^) = 683, 981, 1114, 2914. ^1^H-NMR (300 MHz, CDCl_3_): δ ppm. 7.89–7.83 (4H, m, CH aromatic), 7.82–7.80 (2H, m, CH aromatic), 7.77–7.71 (4H, m, CH aromatic), 7.67–7.61 (3H, m, CH aromatic), 7.57–7.51 (2H, m, CH aromatic), 5.69 (2H, dd, *J *= 6, 12, CH_2_), 3.03 (1H, dt, *J *= 3.9, 6.91, CH), 1.85–1.74 (2H, m, CH_2_), 1.65–1.64 (2H, m, CH_2_), 1.63–1.57 (1H, m, CH), 1.53–1.38 (2H, m), 0.90 (3H, s, CH_3_), 0.72 (3H, s, CH_3_), 0.51 (3H, s, CH_3_) ^13^C-NMR (75 MHz, CDCl_3_): δ ppm. 135.3, 135.3, 134.0 (3C), 133.9, 132.0, 131.9, 130.4, 130.3 (3C), 128.5 (3C), 128.4 (3C), 116.5 (d, *J* = 85), 76.3, 49.0, 48.6, 41.5, 41.4, 39.2, 26.2, 21.0, 20.2, 19.8; ^31^P (202 MHz, CDCl_3_): δ ppm 19.00. EIMS = 430 (M^+^-I), 277.2 (7.7%), 262.2 (55.9%), 183.1 (100%), 167.1 (49.8%), 152.1 (14.8%), 108.0 (13.4%), 91.0 (43.9%).

#### *tert*-Butoxymethyltriphenylphosphonium iodide (**2j**)

Yellowish thick oil, yield = 77%, IR: υ (cm^−1^) = 690, 713, 1127, 1295, 1405, 2799. ^1^H-NMR (400 MHz, MeOD): δ ppm. 7.91–7.90 (2H, m, CH aromatic), 7.89–7.86 (4H, m, CH aromatic), 7.83–7.75 (3H, m, CH aromatic), 7.34–7.31 (4H, m, CH aromatic), 7.25–7.23 (2H, m, CH aromatic), 5.45 (2H, dd, *J *= 1.6, 16.8, CH_2_), 0.047 (9H, s, CH_3_). ^13^C-NMR (75 MHz, CDCl_3_): δ ppm. 136.69 (3 carbons), 136.63, 135.39, 135.27, 134.81, 134.76 (3 carbons), 133.66, 133.13, 132.67, 131.13, 131.09, 129.79 (3 carbons), 117.69, 89.54, 28.76 (3 carbons). ^31^P (202 MHz, CDCl_3_): δ ppm 18.98. EIMS = 349 (M^+^-I), 277.2 (100%), 262.2 (67.6%), 201.1 (24.5%), 183.1 (54.9%), 152.1 (11.4%), 108.0 (10.9%), 77.0 (9.8%).

### General method for synthesis of vinyl ethers **3a**–**e**

In a two neck round bottom flask *n*-BuLi (1.5 eq) was added to stirred solution of phosphonium iodide **2** (1 eq) in THF at − 78 °C and mixture was allowed to stir under argon. After 20 min solution of aldehyde (1 eq) in THF was added drop wise at the same temperature and reaction mixture was allowed to stir for further 4 h allowing the temperature to come to room temperature slowly. Reaction was monitored on TLC, after completion reaction was quenched with methanol and solvent was evaporated under reduced pressure. Products were purified on silica gel column by combinations of ethyl acetate and pet ether as eluent.

#### 2-Ethoxyethenyl benzene (**3a**–**a′**, *mixture of cis and trans isomers*) [38]

^1^H NMR (400 MHz, CDCl_3_) δ ppm. 8.00–7.97 (1H, m), 7.62–7.56 (1H, m), 7.50–7.46 (1 H, m), 7.32–7.25 (5H, m), 7.17–7.13 (1H, m), 7.01 (0.76H, d, *J* = 12.9), 6.23 (0.26H, d, *J* = 7.0), 5.86 (0.73H, d, *J* = 12.9), 5.24 (0.27H, d, *J* = 8.0), 4.01 (0.56H, q, *J* = 7.2), 3.92 (1.5 H, q, *J* = 7.3), 1.46–1.35 (6H, m); HRMS GC/MS calculated for C_10_H_12_O: 148.0883; found 148.0879.

#### 1-Chloro-4[2-ethoxyethenyl]benzene (**3b**–**b′**, *mixture of cis and trans isomers*) [39]

^1^H NMR (400 MHz, CDCl_3_) δ ppm. 7.51–7.15 (4H, m), 6.94 (0.69H, d, *J* = 12.0), 6.37 (0.31H, d, *J* = 8.0), 5.83 (0.71H, d, *J* = 12.0), 5.69 (0.29H, d, *J* = 7.4), 3.95 (0.62H, q, *J* = 7.4), 3.86 (1.43H, q, *J* = 7.2), 1.34–1.26 (6H, m); HRMS GC/MS calculated for C_10_H_11_OCl: 182.0493; found 182.0501.

#### 1-Bromo-4[2-ethoxyethenyl]benzene (**3c**–**c′**, *mixture of cis and trans isomers*) [42, 62]

^1^H NMR (400 MHz, CDCl_3_) δ = 7.31–7.21 (4H, m), 7.01 (0.73H, d, *J* = 12.8), 6.51 (0.29H, d, *J* = 7.1), 5.83 (0.70H, d, *J* = 12.8), 5.69 (0.31H, d, *J* = 7.3), 4.12 (1.42H, q, *J* = 7.2), 3.93 (0.63H, q, *J* = 7.5), 1.45–1.37 (6H, m); HRMS GC/MS calculated for C_10_H_11_OBr: 225.9988; found 225.9988.

#### 1-[(1*E* & *Z*)-2-ethoxyethenyl]-4-methoxybenzene (**3d**–**d′**) [42, 62]

(*Mixture of cis and trans isomers*) ^1^H NMR (400 MHz, CDCl_3_) δ ppm 7.57–7.15 (4H, m), 6.79 (0.63H, d, *J* = 13.0), 6.13 (0.37H, d, *J* = 8.0), 6.10 (0.64H, d, *J* = 12.9), 5.65 (0.38H, d, *J* = 7.8), 3.89 (4H, q, *J* = 7.5), 1.43 (6H, m). HRMS GC/MS calculated for C_11_H_14_O_2_: 178.0988; found 178.0991.

#### (*E*)-(2-((2-isopropyl-5-methylcyclohexyl)oxy)vinyl)benzene (**3e′**) [40]

Colorless oil; yield = 43%, ^1^H NMR (CDCl_3_, 400 MHz): δ ppm 7.28–7.22 (4H, m), 7.15–7.11 (1H, m), 6.92 (1H, d, *J* = 12.6), 5.93 (1H, d, *J* = 12.6), 3.62 (1H, td, *J* = 4.3), 2.21–2.10 (2H, m), 1.72–1.71 (1H, m), 1.69–1.68 (1H, m), 1.58–1.52 (1H, m), 1.49–1.39 (2H, m), 1.11–1.01 (2H, m), 0.95 (3H, d, *J* = 6.16), 0.94 (3H, d, *J* = 6.6), 0.82 (3H, d, *J* = 6.9); ^13^C NMR (CDCl_3_, 100 MHz): δ ppm 147.5, 136.7, 128.6 (2C), 125.4 (2C), 124.9, 107.0, 81.6, 47.8, 41.4, 34.3, 31.5, 25.8, 23.4, 22.1, 20.7, 16.4. HRMS GC/MS calculated for C_18_H_26_O; 258.1984, found; 258.1987.

### General method for carbon homologation in aldehydes

In a two neck round bottom flask containing phosphonim iodide **2a** (1 eq) in dry THF (5 mL), *n*-BuLi (1.5 eq) was added dropwise at − 78 °C and mixture was allowed to stir for 30 min. Solution of aldehyde (1 eq) in THF was added dropwise to the phosphorene reaction mixture and further allowed to stir for 5 h. After acidic hydrolysis, crude product was extracted with EtOAc (10 mL × 2). Combined extract was dried over Na_2_SO_4_, concentrated and purified on preparative TLC (silica gel) to obtain higher analogue of aldehydes (see Additional file 1).

### General procedure for asymmetric reduction reaction

In a two-neck round bottom flask, acetophenone (1.5 mmol), NaBH_4_ (2.25 mmol) along with iodide salt **2g** (10 mol%) was taken in methanol (5 mL). Reaction mixture was stirred for 2 h at room temperature. The reaction progress was monitored by TLC and after completion; the mixture was quenched with water and extracted EtOAc (2 × 3 mL). Combined organic layer was dried over MgSO_4_ and the solvent was evaporated under reduced pressure to afford the corresponding (*R*)-1-phenylethanol (92% yield, 4% ee). Enantiomeric excess (ee) was calculated on HPLC using chiral cellulose OD-H column, hexane/i-PrOH, 95:5, flow rate 1 mL/min (see Additional file 1).

## Additional file

**Additional file 1.** General method for synthesis of *Bis*-alkoxy methanes.

**Additional file 2.** Carbon Homologation in aldehydes.

**Additional file 3.** Asymmetric reduction of acetophenone.

**Additional file 4.** Crystallography data for(*S*)-*sec*-Butoxymethyltriphenylphosphonium iodide.

**Additional file 5.** Specimen NMR Spectra of alkoxymethyltriphenylphosphonium iodides.

**Additional file 6.** Specimen NMR Spectrum of vinyl ether.
