# Supplementary material for: Facile synthesis of α-alkoxymethyltriphenylphosphonium iodides: new application of PPh3/I2
Source: Chem Cent J. 2018 May 17;12:62. doi: 10.1186/s13065-018-0421-6 (PMC5957017; doi:10.1186/s13065-018-0421-6)
Supplement: Supplementary file 1 — Additional file 1. General method for synthesis of Bis-alkoxy methanes. [file 13065_2018_421_MOESM1_ESM.docx]

# Additional file 1

# General method for synthesis of *Bis*-alkoxy methanes (1)

Alcohol (5g) was dissolved in 100-120 ml of toluene in a two neck round bottom flask along with para-formaldehyde (6g) and few crystals of *p*-TSA. Apparatus was set with dean stark distillation head that allowed the azeotropic removal of water. The reaction was monitored by TLC. After completion, the reaction mixture was cooled to room temperature and excess toluene was removed under reduced pressure. The crude product was extracted with 3 x 20 mL EtOAc. Combined organic layer was washed with brine and NaHCO_3_ solution, dried over anhydrous MgSO_4_ and evaporated under reduced pressure. The crude product was purified by distillation or column chromatography as per requirement.

**Scheme** : Synthesis of *bis*-alkoxy methanes (**1**) from alcohols

***Bis*-*n*-butoxy methane** (**1a**) [S1]: Light yellow liquid, bp 178-181 ^o^C (179-180 ^o^C Lit.), yield = 68%. IR: υ (cm^-1^) 1206, 2973, 1358. ^1^H-NMR (300 MHz, CDCl_3_): δ ppm 4.59 (2H, s, CH_2_), 3.42 (4H, t, *J* = 6.4, OCH_2_), 1.41-1.45 (2H, m, CH_2_), 1.51-1.53 (2H, m, CH_2_), 0.81 (3H, t, *J* = 7.4, CH_3_). ^13^C-NMR (75 MHz, CDCl_3_): δ ppm 97, 69, 33, 19, 15. GCMS: 160 (M^+^), 87, 57, 41, 29.

***Bis*-methoxy methane** (**1b**) [S2]: Colorless liquid, bp 42-45 ^o^C, yield = 74%, IR: υ (cm^-1^) 2976, 1374, 1222. ^1^H-NMR (300MHz, CDCl_3_): δ ppm 4.57 (2H, s, CH_2_), 3.37 (6H, s, OCH_3_). ^13^C-NMR (75 MHz, CDCl_3_): δ ppm 97, 56. GCMS: 76 (M^+^), 75, 45, 29, 15.

***Bis*(benzoxy)methane** (**1d**) [S3]: Yellow thick liquid, yield = 85%, IR: υ (cm^-1^) 1065, 3056, 2938, 1356. ^1^H-NMR (300MHz, CO(CD_3_)_2_): δ ppm 7.40-7.28 (10H, m, CH), 4.65 (6H, s, CH_2_). ^13^C-NMR: (75MHz, CO(CD_3_)_2_): δ ppm. 138, 129, 95, 70. GCMS: 228 (M^+^), 121, 107, 92, 77, 51.

**Diphenethoxymethane** (**1e**): Light yellow thick liquid, yield = 82%, IR: υ (cm^-1^) 3027, 2938, 1111, 1366. ^1^H-NMR (300MHz, CO(CD_3_)_2_): δ ppm 7.40-7.29 (10H, m, CH), 4.52 (2H, s, CH_2_), 3.58 (2H, t, *J* =6.2 Hz, CH_2_), 2.81 (2H, t, *J* =6.9 Hz, CH_2_). ^13^C-NMR: (75MHz, CO(CD_3_)_2_): δ ppm. 138, 129, 97, 73, 37. GCMS: 257 (M^+^), 165, 152, 135, 121, 105, 91, 77, 65, 51.

***Bis* ((*S*)-sec-butoxy)methane** (**1f**): Lemon yellow liquid, bp 168 ^o^C, $\left[\alpha\right]_{D}^{25}$= -6.23 (c = 5mg/15mL CHCl_3_). yield = 77%, IR: υ (cm^-1^) 1218, 2969 cm^-1^, 1378. ^1^H-NMR (300MHz, CDCl_3_): δ ppm. 4.68 (2H, s, CH_2_), 3.27 (2H, m, CH), 2.24-2.14 (4H, m, CH_2_), 1.59 (6H, d, *J =* 6.0 Hz, CH_3_), 0.93 (6H, t*, J =* 3.0 Hz, CH_3_), ^13^C-NMR (75MHz, CDCl_3_): δ ppm 9.7, 19.4, 32.0, 73.4, 90.9. GCMS: 160 (M^+^), 101, 87, 57, 45, 41, 29.

***Bis*((1,3,3-trimethylbicyclo[2.2.1]heptan-2-yl)oxy)methane** (**1g**): Yellow thick liquid, $\left[\alpha\right]_{D}^{25}$= +1.73 (c = 5mg/20mL CHCl_3_). yield = 85%, IR: υ (cm^-1^) 2847, 1366, 1115. ^1^H-NMR (300MHz, CDCl_3_): δ ppm. 4.62 (2H, s, CH_2_), 3.18 (2H, d, *J* = 1.5 Hz, CH), 1.78-1.72 (4H, m, CH_2_), 1.70-1.69 (2H, m, CH), 1.63-1.62 (4H, m, CH_2_), 1.45-1.36 (4H, m, CH_2_), 1.04 (6H, s, CH_3_), 0.90 (6H, s, CH_3_), 0.87 (6H, s, CH_3_). ^13^C-NMR: (75MHz, CDCl_3_): δ ppm. 91.07, 85.53, 51.98, 46.30, 43.67, 40.21, 36.45, 30.63, 19.81, 17.88, 11.22. GCMS: 320 (M^+^), 183, 168, 153, 137, 95.

**(((1*S*, 2*R*)-2-isopropyl-5-methyl cyclohexyl)oxy)(((1*S*, 2*S*)-2-isopropyl-5-methylcyclohexyl) oxy)methane** (**1h**) [S4]: White crystals, mp 61^o^C, $\left[\alpha\right]_{D}^{25}$= -8.40 (c = 5mg/15mL CHCl_3_). yield = 80%, IR: υ (cm^-1^) 2976, 1374, 1181. ^1^H-NMR (300MHz, CDCl_3_): δ ppm. 4.83 (2H, s, CH_2_), 3.32 (2H, dt, *J* = 4.5, 10.8Hz, CH), 2.24-2.14 (4H, m, CH_2_), 1.67-1.59 (4H, m, CH_2_), 1.42-1.35 (2H, m, CH), 1.34-1.28 (2H, m, CH), 1.21-1.16 (2H, m, CH) , 1.01-0.93 (4H, m, CH_2_), 0.93 (6H, d*, J =* 3.0 Hz, CH_3_), 0.91 (6H, d, *J =* 3.6 Hz, CH_3_), 0.79 (6H, d, *J* = 6.9 Hz, CH_3_). ^13^C-NMR (75MHz, CDCl_3_): δ ppm. 95.34, 88.92, 48.60, 42.45, 34.38, 31.63, 29.71, 23.10, 22.30, 21.21, 16.07. GCMS: 324 (M+) 185, 169, 156, 139, 125.

***Bis*(((2R)-1,7,7-trimethyl bicyclo[2.2.1] heptan-2-yl)oxy)methane** (**1i**): White shiny crystals, mp = 156ºC, $\left[\alpha\right]_{D}^{25}$= +2.13 (c = 5mg/15mL CHCl_3_). yield = 78%, IR: υ (cm^-1^) 2831, 1378, 1127. ^1^H-NMR: (300MHz, CDCl_3_) δ ppm. 4.69-4.60 (2H, dd, *J* = 2.1, 3.9 Hz, CH_2_), 3.62-3.53 (2H, dt, *J* = 3.9, 8.7, CH), 1.95-1.92 (4H, m, CH_2_), 1.68-1.62 (4H, m, CH_2_), 1.22-1.19 (4H, m, CH_2_), 1.03-1.01 (4H, m, CH_2_), 0.98 (6H, d, *J* = 9 Hz, CH_3_) 0 .87 (6H, s, CH_3_) 0.82 (6H, s*,* CH_3_). ^13^C-NMR: (75MHz, CDCl_3_): δ ppm. 89.04, 83.72, 48.98, 46.30, 44.97, 39.21, 34.45, 26.63, 19.81, 18.86, 11.2. GCMS: 320 (M^+^), 183, 168, 153, 137, 123, 95, 81, 69, 55.

***Bis*-*tert*-butoxy methane** (**1j**) [S5]: Colorless thick liquid, bp 145-148 ^o^C (148 ^o^C Lit.), yield = 65%, IR: υ (cm^-1^) 1222, 2976, 1366. ^1^H-NMR (300MHz, CDCl_3_): δ ppm. 4.8 (2H, s, CH_2_), 1.16 (18H, s, CH_3_). ^13^C-NMR (75MHz, CDCl_3_): δ ppm 87, 78, 29. GCMS: 160 (M^+^), 101, 87, 57, 45, 41, 29.

**References**

[S1] B. Burczyk, [*J. fur praktische Chemie*](https://www.researchgate.net/journal/1521-3897_Journal_fuer_praktische_Chemie)*.*, 1980, **322**, 173-176.

[S2] M. Berliner and K. Belecki*,* [*Org. Synth.*](https://en.wikipedia.org/wiki/Organic_Syntheses)2007, **84**, 102.

[S3] J.H. Zaidi, K. M. Khan, S. Mir, N. I. Gunjial and M. Arfan, *Letters in Organic Chemistry****,* 2008,** 05, 125-127.

[S4] S. Mumtaz, S. W. Khan, J. H. Zaidi, A. Iqbal, Z. M. Cheema, K. M. Khan and S. Parveen, *Letters in Organic Chemistry****,*** 2013**, 10**, 578-583.

[S5] (a) [M. John Perkins](http://pubs.rsc.org/en/results?searchtext=Author%3AM.%20John%20Perkins) and [B. P. Roberts](http://pubs.rsc.org/en/results?searchtext=Author%3ABrian%20P.%20Roberts), *J. Chem. Soc., Perkin Trans.* 1975, **2,** 77-84. (b) I. Jansson; *Suomen Kem*.,1964, **37B**, 19.
