# Supplementary material for: Facile synthesis of α-alkoxymethyltriphenylphosphonium iodides: new application of PPh3/I2
Source: Chem Cent J. 2018 May 17;12:62. doi: 10.1186/s13065-018-0421-6 (PMC5957017; doi:10.1186/s13065-018-0421-6)
Supplement: Supplementary file 2 — Additional file 2. Carbon Homologation in aldehydes. [file 13065_2018_421_MOESM2_ESM.docx]

**Additional file 2.**

**Carbon Homologation in aldehydes**

**2-phenylacetaldehyde (4a)**: Light yellow liquid, Yield = 72%, ^1^H-NMR (300 MHz, MeOD): δ ppm. 9.72 (1H, s, aldehydic), 7.39-7.20 (5H, m, aromatic), 3.67 (2H, s, CH_2_).

**1-butanal (4b):** Colorless liquid, Yield = 71%, ^1^H-NMR (300MHz, MeOD): δ ppm.8.79 (1H, s, aldehydic), 2.33 (2H, t, *J* = 7.5 Hz, CH_2_), 1.45-1.21 (2H, m, CH_2_), 0.86 (2H, t, *J* = 4.2 Hz, CH_3_).

**1-pentanal (4c):** Colorless liquid, Yield = 73%, ^1^H-NMR (300MHz, MeOD): δ ppm. 9.27 (1H, s, aldehydic), 2.32 (2H, t, *J* = 6 Hz, CH_2_), 1.60-1.53 (2H, m, CH_2_), 1.36-1.26 (2H, m, CH_2_), 0.86 (3H, t, *J* = 4.2 Hz, CH_3_).

**1-hexanal (4d):** Colorless liquid, Yield = 70%, ^1^H-NMR (300MHz, MeOD): δ ppm. 9.63 (1H, s, aldehydic), 2.41 (2H, dt, *J* = 6.2, 4.1 Hz, CH_2_), 1.63-1.56 (2H, m, CH_2_), 1.30-1.29 (4H, m, 2xCH_2_), 0.89 (3H, t, *J* = 5.3 Hz, CH_3_).
