# Supplementary material for: Facile synthesis of α-alkoxymethyltriphenylphosphonium iodides: new application of PPh3/I2
Source: Chem Cent J. 2018 May 17;12:62. doi: 10.1186/s13065-018-0421-6 (PMC5957017; doi:10.1186/s13065-018-0421-6)
Supplement: Supplementary file 3 — Additional file 3. Asymmetric reduction of acetophenone. [file 13065_2018_421_MOESM3_ESM.docx]

**Additional file 3.**

**Asymmetric reduction of acetophenone**

**(*R*)-1-phenylethanol (5):** Yield = 92%, $\left[\alpha\right]_{D}^{25}$= -19.3 (*c*0.30, MeOH). ^1^H NMR (CDCl_3_, 500 MHz): δ ppm. 7.37–7.21 (5H, m, Ar), 4.87–4.83 (1H, q, CH), 1.91 (1H, s, OH), 1.49–1.45 (3H, d, *J* = 6.0 Hz, CH_3_). GC-MS: m/z, 122 (M^+^), 107 , 77 . HPLC: Chiral cellulose OD-H column, hexane/i-PrOH, 95:5, flow rate 1 mL/min, t_R_ (major) = 9.38 min; t_R_ (minor) = 11.71 min.
