# Supplementary material for: Facile synthesis of α-alkoxymethyltriphenylphosphonium iodides: new application of PPh3/I2
Source: Chem Cent J. 2018 May 17;12:62. doi: 10.1186/s13065-018-0421-6 (PMC5957017; doi:10.1186/s13065-018-0421-6)
Supplement: Supplementary file 4 — Additional file 4. Crystallography data for(S)-sec-Butoxymethyltriphenylphosphonium iodide. [file 13065_2018_421_MOESM4_ESM.docx]

**Additional file 4.**

**Crystallography data for (*S*)-*sec*-Butoxymethyltriphenylphosphonium iodide (2f)**


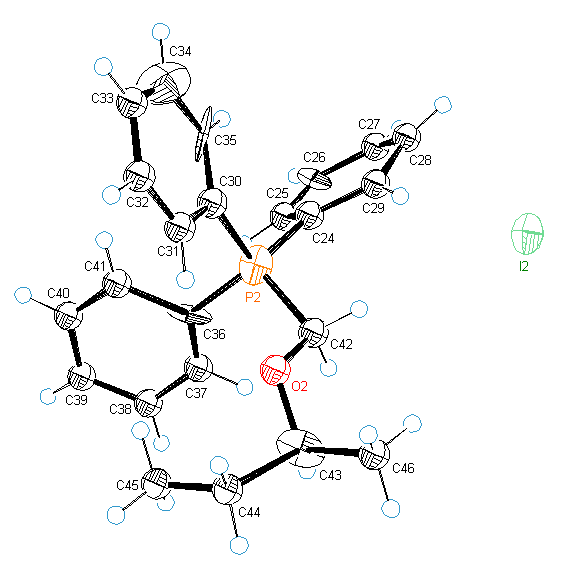
(CCDC 1537362)

**Table1.** Crystal data and structure refinement for (*S*)-(*sec*-butoxymethyl)iodotriphenylphosphorane (4f)

Identification code sbi_0m

Empirical formula C23 H26 I O P

Formula weight 476.31

Temperature 100(2) K

Wavelength 1.54178 A

Crystal system, space group Triclinic, P1

Unit cell dimensions a = 9.464(8) A alpha = 91.39(6) deg.

b = 9.903(7) A beta = 101.66(6) deg.

c = 12.368(9) A gamma = 102.42(7) deg.

Volume 1105.9(14) A^3

Z, Calculated density 2, 1.430 Mg/m^3

Absorption coefficient 12.111 mm^-1

F(000) 480

Crystal size 0.31 x 0.19 x 0.11 mm

Theta range for data collection 3.66 to 47.45 deg.

Limiting indices -9<=h<=9, -9<=k<=9, -11<=l<=11

Reflections collected / unique 10504 / 3930 [R(int) = 0.2942]

Completeness to theta = 47.45 99.5 %

Max. and min. transmission 0.3493 and 0.1172

Refinement method Full-matrix least-squares on F^2

Data / restraints / parameters 3930 / 3 / 469

Goodness-of-fit on F^2 1.515

Final R indices [I>2sigma(I)] R1 = 0.1526, wR2 = 0.3577

R indices (all data) R1 = 0.1595, wR2 = 0.3666

Absolute structure parameter 0.04(3)

Largest diff. peak and hole 3.332 and -2.621 e.A^-3

**Table 2.** Atomic coordinates ( x 10^4) and equivalent isotropic displacement parameters (A^2 x 10^3) for sbi_0m. U(eq) is defined as one third of the trace of the orthogonalized Uij tensor.

________________________________________________________________

x y z U(eq)

________________________________________________________________

P(1) 5378(10) 2518(10) 7664(8) 35(3)

P(2) 3151(12) 7481(10) 2169(8) 40(3)

I(1) 11433(1) 8247(1) 5752(1) 42(1)

I(2) 7056(1) 11737(1) 4134(1) 41(1)

O(1) 6790(30) 460(30) 7710(20) 55(9)

O(2) 1800(30) 9500(30) 2106(19) 37(7)

C(1) 4040(50) 4470(40) 6500(40) 49(12)

C(2) 2810(70) 4810(40) 5820(30) 67(16)

C(3) 1460(70) 3960(50) 5510(40) 70(20)

C(4) 1420(40) 2710(50) 5800(40) 50(13)

C(5) 2530(40) 2200(40) 6550(20) 32(9)

C(6) 3950(40) 3080(40) 6860(30) 47(11)

C(7) 4950(30) 2310(30) 8990(20) 18(8)

C(8) 3910(40) 2930(50) 9360(40) 47(13)

C(9) 3630(40) 2860(40) 10410(30) 35(11)

C(10) 4460(40) 2160(40) 11120(40) 70(20)

C(11) 5520(50) 1450(40) 10880(40) 62(15)

C(12) 5800(40) 1570(40) 9700(30) 48(14)

C(13) 7110(40) 3850(30) 7850(40) 39(11)

C(14) 7520(50) 4810(40) 8700(30) 45(11)

C(15) 8710(40) 5920(50) 8720(50) 64(16)

C(16) 9450(40) 5980(50) 7930(30) 56(15)

C(17) 9070(40) 5050(40) 7040(30) 44(10)

C(18) 7930(40) 3870(40) 6910(30) 39(10)

C(19) 5500(50) 900(30) 7020(20) 35(11)

C(20) 7760(40) -110(40) 7160(40) 43(10)

C(21) 6740(40) -1690(40) 6670(30) 62(13)

C(22) 8920(70) -370(40) 7920(40) 86(19)

C(23) 9930(40) 1150(60) 8500(30) 110(20)

C(24) 4720(40) 6980(40) 3050(30) 27(9)

C(25) 4430(50) 5670(60) 3350(30) 56(13)

C(26) 5510(50) 5150(50) 3970(40) 63(16)

C(27) 6890(50) 6100(50) 4300(30) 60(20)

C(28) 7320(40) 7510(40) 3970(30) 48(13)

C(29) 6070(60) 7900(40) 3450(40) 65(16)

C(30) 3490(50) 7800(40) 760(40) 56(13)

C(31) 2730(40) 8510(40) 60(30) 48(12)

C(32) 2860(40) 8520(40) -970(40) 41(11)

C(33) 3900(50) 7940(40) -1370(30) 58(13)

C(34) 4600(70) 7310(50) -660(50) 80(20)

C(35) 4470(60) 7140(40) 470(30) 54(14)

C(36) 1510(40) 6260(40) 2040(30) 49(13)

C(37) 740(30) 6030(30) 2800(40) 36(10)

C(38) -500(40) 4930(40) 2820(30) 40(10)

C(39) -1000(50) 3970(40) 1890(50) 70(20)

C(40) -200(50) 4160(30) 1050(30) 41(12)

C(41) 1020(30) 5260(40) 1090(30) 40(10)

C(42) 2840(50) 9010(40) 2830(40) 62(14)

C(43) 950(40) 10260(50) 2690(30) 52(12)

C(44) -500(30) 10160(50) 1770(30) 67(15)

C(45) -1390(50) 8810(30) 1330(30) 100(20)

C(46) 1810(60) 11630(30) 3160(40) 81(17)

________________________________________________________________

**Table 3.** Bond lengths [A] and angles [deg] for sbi_0m.

_____________________________________________________________

P(1)-C(6) 1.71(5)

P(1)-C(7) 1.77(3)

P(1)-C(19) 1.80(3)

P(1)-C(13) 1.84(3)

P(2)-C(36) 1.73(4)

P(2)-C(24) 1.83(4)

P(2)-C(42) 1.81(5)

P(2)-C(30) 1.85(4)

O(1)-C(20) 1.43(5)

O(1)-C(19) 1.50(5)

O(2)-C(42) 1.37(6)

O(2)-C(43) 1.49(5)

C(1)-C(2) 1.40(8)

C(1)-C(6) 1.45(5)

C(1)-H(1A) 0.9500

C(2)-C(3) 1.35(8)

C(2)-H(2A) 0.9500

C(3)-C(4) 1.29(6)

C(3)-H(3A) 0.9500

C(4)-C(5) 1.44(6)

C(4)-H(4A) 0.9500

C(5)-C(6) 1.41(5)

C(5)-H(5) 0.9500

C(7)-C(12) 1.41(6)

C(7)-C(8) 1.41(5)

C(8)-C(9) 1.38(5)

C(8)-H(8A) 0.9500

C(9)-C(10) 1.36(7)

C(9)-H(9A) 0.9500

C(10)-C(11) 1.42(6)

C(10)-H(10A) 0.9500

C(11)-C(12) 1.53(6)

C(11)-H(11) 0.9500

C(12)-H(12A) 0.9500

C(13)-C(14) 1.34(5)

C(13)-C(18) 1.52(5)

C(14)-C(15) 1.39(6)

C(14)-H(14) 0.9500

C(15)-C(16) 1.30(6)

C(15)-H(15A) 0.9500

C(16)-C(17) 1.36(5)

C(16)-H(16A) 0.9500

C(17)-C(18) 1.39(5)

C(17)-H(17A) 0.9500

C(18)-H(18) 0.9500

C(19)-H(19A) 0.9900

C(19)-H(19B) 0.9900

C(20)-C(22) 1.36(7)

C(20)-C(21) 1.68(6)

C(20)-H(20A) 1.0000

C(21)-H(21A) 0.9800

C(21)-H(21B) 0.9800

C(21)-H(21C) 0.9800

C(22)-C(23) 1.65(6)

C(22)-H(22A) 0.9900

C(22)-H(22B) 0.9900

C(23)-H(23A) 0.9800

C(23)-H(23B) 0.9800

C(23)-H(23C) 0.9800

C(24)-C(25) 1.34(6)

C(24)-C(29) 1.39(6)

C(25)-C(26) 1.36(8)

C(25)-H(25A) 0.9500

C(26)-C(27) 1.41(7)

C(26)-H(26A) 0.9500

C(27)-C(28) 1.47(6)

C(27)-H(27A) 0.9500

C(28)-C(29) 1.36(7)

C(28)-H(28) 0.9500

C(29)-H(29A) 0.9500

C(30)-C(35) 1.35(7)

C(30)-C(31) 1.33(6)

C(31)-C(32) 1.30(6)

C(31)-H(31) 0.9500

C(32)-C(33) 1.41(6)

C(32)-H(32A) 0.9500

C(33)-C(34) 1.25(7)

C(33)-H(33) 0.9500

C(34)-C(35) 1.43(7)

C(34)-H(34A) 0.9500

C(35)-H(35) 0.9500

C(36)-C(37) 1.30(5)

C(36)-C(41) 1.45(5)

C(37)-C(38) 1.42(5)

C(37)-H(37A) 0.9500

C(38)-C(39) 1.41(6)

C(38)-H(38) 0.9500

C(39)-C(40) 1.39(7)

C(39)-H(39A) 0.9500

C(40)-C(41) 1.40(5)

C(40)-H(40A) 0.9500

C(41)-H(41A) 0.9500

C(42)-H(42A) 0.9900

C(42)-H(42B) 0.9900

C(43)-C(46) 1.46(6)

C(43)-C(44) 1.58(5)

C(43)-H(43A) 1.0000

C(44)-C(45) 1.45(5)

C(44)-H(44A) 0.9900

C(44)-H(44B) 0.9900

C(45)-H(45A) 0.9800

C(45)-H(45B) 0.9800

C(45)-H(45C) 0.9800

C(46)-H(46A) 0.9800

C(46)-H(46B) 0.9800

C(46)-H(46C) 0.9800

C(6)-P(1)-C(7) 106.6(17)

C(6)-P(1)-C(19) 108.0(18)

C(7)-P(1)-C(19) 111.9(16)

C(6)-P(1)-C(13) 110.5(18)

C(7)-P(1)-C(13) 107.0(17)

C(19)-P(1)-C(13) 112.7(19)

C(36)-P(2)-C(24) 113.1(16)

C(36)-P(2)-C(42) 105(2)

C(24)-P(2)-C(42) 107.9(19)

C(36)-P(2)-C(30) 108.1(18)

C(24)-P(2)-C(30) 111(2)

C(42)-P(2)-C(30) 111(2)

C(20)-O(1)-C(19) 119(3)

C(42)-O(2)-C(43) 112(3)

C(2)-C(1)-C(6) 120(4)

C(2)-C(1)-H(1A) 119.9

C(6)-C(1)-H(1A) 120.0

C(1)-C(2)-C(3) 125(4)

C(1)-C(2)-H(2A) 117.3

C(3)-C(2)-H(2A) 117.4

C(4)-C(3)-C(2) 113(5)

C(4)-C(3)-H(3A) 123.3

C(2)-C(3)-H(3A) 123.3

C(3)-C(4)-C(5) 128(4)

C(3)-C(4)-H(4A) 115.8

C(5)-C(4)-H(4A) 115.7

C(6)-C(5)-C(4) 117(4)

C(6)-C(5)-H(5) 121.2

C(4)-C(5)-H(5) 121.3

C(5)-C(6)-C(1) 114(4)

C(5)-C(6)-P(1) 121(3)

C(1)-C(6)-P(1) 125(3)

C(12)-C(7)-C(8) 121(3)

C(12)-C(7)-P(1) 116(2)

C(8)-C(7)-P(1) 123(2)

C(9)-C(8)-C(7) 124(3)

C(9)-C(8)-H(8A) 117.9

C(7)-C(8)-H(8A) 117.9

C(10)-C(9)-C(8) 116(3)

C(10)-C(9)-H(9A) 122.0

C(8)-C(9)-H(9A) 122.0

C(9)-C(10)-C(11) 127(4)

C(9)-C(10)-H(10A) 116.5

C(11)-C(10)-H(10A) 116.5

C(10)-C(11)-C(12) 115(4)

C(10)-C(11)-H(11) 122.4

C(12)-C(11)-H(11) 122.4

C(7)-C(12)-C(11) 117(4)

C(7)-C(12)-H(12A) 121.6

C(11)-C(12)-H(12A) 121.6

C(14)-C(13)-C(18) 124(3)

C(14)-C(13)-P(1) 121(3)

C(18)-C(13)-P(1) 115(3)

C(13)-C(14)-C(15) 119(4)

C(13)-C(14)-H(14) 120.5

C(15)-C(14)-H(14) 120.5

C(16)-C(15)-C(14) 120(5)

C(16)-C(15)-H(15A) 120.1

C(14)-C(15)-H(15A) 120.2

C(15)-C(16)-C(17) 124(4)

C(15)-C(16)-H(16A) 118.2

C(17)-C(16)-H(16A) 118.2

C(18)-C(17)-C(16) 124(4)

C(18)-C(17)-H(17A) 118.1

C(16)-C(17)-H(17A) 118.1

C(17)-C(18)-C(13) 110(3)

C(17)-C(18)-H(18) 125.0

C(13)-C(18)-H(18) 125.0

O(1)-C(19)-P(1) 107(2)

O(1)-C(19)-H(19A) 110.2

P(1)-C(19)-H(19A) 110.3

O(1)-C(19)-H(19B) 110.2

P(1)-C(19)-H(19B) 110.2

H(19A)-C(19)-H(19B) 108.5

C(22)-C(20)-O(1) 111(4)

C(22)-C(20)-C(21) 105(3)

O(1)-C(20)-C(21) 103(3)

C(22)-C(20)-H(20A) 112.5

O(1)-C(20)-H(20A) 112.5

C(21)-C(20)-H(20A) 112.5

C(20)-C(21)-H(21A) 109.5

C(20)-C(21)-H(21B) 109.5

H(21A)-C(21)-H(21B) 109.5

C(20)-C(21)-H(21C) 109.5

H(21A)-C(21)-H(21C) 109.5

H(21B)-C(21)-H(21C) 109.5

C(20)-C(22)-C(23) 108(3)

C(20)-C(22)-H(22A) 110.2

C(23)-C(22)-H(22A) 110.2

C(20)-C(22)-H(22B) 110.2

C(23)-C(22)-H(22B) 110.2

H(22A)-C(22)-H(22B) 108.5

C(22)-C(23)-H(23A) 109.5

C(22)-C(23)-H(23B) 109.5

H(23A)-C(23)-H(23B) 109.5

C(22)-C(23)-H(23C) 109.5

H(23A)-C(23)-H(23C) 109.5

H(23B)-C(23)-H(23C) 109.5

C(25)-C(24)-C(29) 123(4)

C(25)-C(24)-P(2) 114(3)

C(29)-C(24)-P(2) 123(3)

C(24)-C(25)-C(26) 120(4)

C(24)-C(25)-H(25A) 119.7

C(26)-C(25)-H(25A) 119.8

C(27)-C(26)-C(25) 115(5)

C(27)-C(26)-H(26A) 122.6

C(25)-C(26)-H(26A) 122.6

C(26)-C(27)-C(28) 128(3)

C(26)-C(27)-H(27A) 116.2

C(28)-C(27)-H(27A) 116.2

C(29)-C(28)-C(27) 109(3)

C(29)-C(28)-H(28) 125.7

C(27)-C(28)-H(28) 125.7

C(28)-C(29)-C(24) 124(4)

C(28)-C(29)-H(29A) 118.1

C(24)-C(29)-H(29A) 118.1

C(35)-C(30)-C(31) 122(4)

C(35)-C(30)-P(2) 115(4)

C(31)-C(30)-P(2) 123(3)

C(32)-C(31)-C(30) 121(4)

C(32)-C(31)-H(31) 119.7

C(30)-C(31)-H(31) 119.8

C(33)-C(32)-C(31) 123(3)

C(33)-C(32)-H(32A) 118.5

C(31)-C(32)-H(32A) 118.5

C(34)-C(33)-C(32) 113(4)

C(34)-C(33)-H(33) 123.5

C(32)-C(33)-H(33) 123.5

C(33)-C(34)-C(35) 129(5)

C(33)-C(34)-H(34A) 115.7

C(35)-C(34)-H(34A) 115.7

C(30)-C(35)-C(34) 113(5)

C(30)-C(35)-H(35) 123.6

C(34)-C(35)-H(35) 123.7

C(37)-C(36)-C(41) 115(3)

C(37)-C(36)-P(2) 125(3)

C(41)-C(36)-P(2) 120(3)

C(36)-C(37)-C(38) 128(4)

C(36)-C(37)-H(37A) 115.9

C(38)-C(37)-H(37A) 115.8

C(39)-C(38)-C(37) 117(4)

C(39)-C(38)-H(38) 121.5

C(37)-C(38)-H(38) 121.6

C(38)-C(39)-C(40) 117(4)

C(38)-C(39)-H(39A) 121.4

C(40)-C(39)-H(39A) 121.4

C(41)-C(40)-C(39) 123(4)

C(41)-C(40)-H(40A) 118.5

C(39)-C(40)-H(40A) 118.5

C(40)-C(41)-C(36) 120(3)

C(40)-C(41)-H(41A) 120.2

C(36)-C(41)-H(41A) 120.2

O(2)-C(42)-P(2) 108(3)

O(2)-C(42)-H(42A) 110.1

P(2)-C(42)-H(42A) 110.1

O(2)-C(42)-H(42B) 110.1

P(2)-C(42)-H(42B) 110.1

H(42A)-C(42)-H(42B) 108.4

C(46)-C(43)-O(2) 113(3)

C(46)-C(43)-C(44) 119(4)

O(2)-C(43)-C(44) 101(3)

C(46)-C(43)-H(43A) 108.0

O(2)-C(43)-H(43A) 108.0

C(44)-C(43)-H(43A) 108.1

C(45)-C(44)-C(43) 119(4)

C(45)-C(44)-H(44A) 107.5

C(43)-C(44)-H(44A) 107.5

C(45)-C(44)-H(44B) 107.6

C(43)-C(44)-H(44B) 107.6

H(44A)-C(44)-H(44B) 107.0

C(44)-C(45)-H(45A) 109.5

C(44)-C(45)-H(45B) 109.5

H(45A)-C(45)-H(45B) 109.5

C(44)-C(45)-H(45C) 109.5

H(45A)-C(45)-H(45C) 109.5

H(45B)-C(45)-H(45C) 109.5

C(43)-C(46)-H(46A) 109.5

C(43)-C(46)-H(46B) 109.5

H(46A)-C(46)-H(46B) 109.5

C(43)-C(46)-H(46C) 109.5

H(46A)-C(46)-H(46C) 109.5

H(46B)-C(46)-H(46C) 109.5

_____________________________________________________________

Symmetry transformations used to generate equivalent atoms:

**Table 4.** Anisotropic displacement parameters (A^2 x 10^3) for sbi_0m. The anisotropic displacement factor exponent takes the form: -2 pi^2 [ h^2 a*^2 U11 + ... + 2 h k a* b* U12 ]

______________________________________________________________________

U11 U22 U33 U23 U13 U12

_______________________________________________________________________

P(1) 37(6) 29(6) 36(6) 6(5) 10(5) 1(4)

P(2) 48(6) 37(7) 31(6) 9(5) 5(5) 7(5)

I(1) 51(2) 39(2) 34(2) 6(1) 6(1) 3(1)

I(2) 50(2) 36(2) 32(2) 5(1) 5(1) 3(1)

O(1) 68(19) 33(17) 52(18) 28(14) 7(16) -11(15)

O(2) 30(14) 58(18) 27(14) -33(13) -4(12) 28(13)

C(1) 80(30) 13(19) 80(30) 43(19) 60(30) 40(20)

C(2) 160(50) 0(20) 50(30) 30(18) 0(30) 60(30)

C(3) 140(50) 30(30) 40(30) -20(20) 40(30) -40(30)

C(4) 30(20) 40(30) 70(30) -40(20) -10(20) 12(19)

C(5) 30(20) 40(20) 3(15) 11(14) -36(15) 9(18)

C(6) 30(20) 50(30) 50(30) -10(20) 7(19) -13(18)

C(7) 13(16) 33(19) 13(16) 21(14) -12(13) 30(15)

C(8) 20(20) 60(30) 70(40) 30(20) 10(20) 40(20)

C(9) 11(16) 90(30) 20(20) 28(19) -4(15) 58(19)

C(10) 20(20) 40(30) 150(50) -60(30) 70(30) -50(20)

C(11) 70(30) 40(30) 40(30) -10(20) 10(20) -50(20)

C(12) 30(20) 30(20) 70(30) -50(20) 40(20) -35(18)

C(13) 22(19) 0(16) 90(30) -13(19) 20(20) -7(14)

C(14) 80(30) 20(20) 20(20) 13(18) -10(20) 10(20)

C(15) 20(20) 50(30) 100(40) 0(20) -20(20) -20(20)

C(16) 40(20) 110(40) 10(20) 20(20) 40(20) -20(20)

C(17) 40(20) 40(30) 50(30) 0(20) -9(18) 20(20)

C(18) 40(20) 50(20) 30(20) 24(17) 27(18) 1(19)

C(19) 90(30) 20(20) 6(18) 6(16) -12(17) 60(20)

C(20) 40(20) 30(20) 70(30) 0(20) 20(20) 36(18)

C(21) 60(20) 110(40) 20(20) -10(20) 25(19) 0(20)

C(22) 170(60) 20(20) 90(40) 30(20) 40(40) 80(30)

C(23) 20(20) 270(70) 70(30) -30(30) 80(20) 30(30)

C(24) 50(30) 5(18) 40(20) 6(16) 28(19) 15(18)

C(25) 30(20) 120(40) 2(18) -20(20) -25(17) 10(20)

C(26) 30(20) 90(40) 50(30) -40(30) 10(20) -20(20)

C(27) 90(40) 120(50) 40(20) 20(30) 0(20) 150(40)

C(28) 30(20) 70(30) 80(30) 60(20) 23(19) 80(20)

C(29) 90(40) 0(19) 100(40) -20(20) 40(30) -10(20)

C(30) 80(30) 30(20) 60(30) -30(20) 30(30) -20(20)

C(31) 60(30) 60(30) 40(30) 60(20) 10(20) 50(20)

C(32) 30(20) 40(20) 50(30) 20(20) -24(19) 16(18)

C(33) 110(30) 80(30) 0(15) 55(17) -10(18) 70(20)

C(34) 120(50) 40(30) 100(50) 10(30) 70(40) -10(30)

C(35) 110(40) 30(30) 10(20) -4(18) 0(20) -10(30)

C(36) 30(20) 110(40) 10(20) 40(20) 9(19) 50(20)

C(37) 0(15) 30(20) 80(30) -20(19) -1(18) 17(15)

C(38) 40(20) 70(30) 30(20) 0(20) 41(18) 10(20)

C(39) 80(30) 0(20) 110(50) 30(20) -60(30) 10(19)

C(40) 100(40) 0(18) 20(20) 14(15) 20(20) 0(20)

C(41) 21(19) 60(30) 40(20) -10(20) 40(18) -7(18)

C(42) 50(30) 50(30) 60(30) -10(20) 10(20) -50(20)

C(43) 50(20) 70(30) 50(20) 30(20) 11(19) 20(20)

C(44) 7(16) 160(40) 20(20) -10(20) 3(15) -10(20)

C(45) 120(40) 17(19) 70(30) 13(19) -100(30) -60(20)

C(46) 130(40) 0(19) 90(40) 10(20) -20(30) 10(20)

_______________________________________________________________________

**Table 5.** Hydrogen coordinates ( x 10^4) and isotropic displacement parameters (A^2 x 3) for sbi_0m.

_____________________________________________________________

x y z U(eq)

________________________________________________________________

H(1A) 4937 5156 6720 58

H(2A) 2939 5714 5563 81

H(3A) 623 4245 5109 88

H(4A) 540 2043 5490 59

H(5) 2318 1304 6814 39

H(8A) 3368 3437 8849 57

H(9A) 2910 3277 10630 42

H(10A) 4314 2148 11857 87

H(11) 6011 929 11403 74

H(12A) 6523 1158 9471 58

H(14) 6995 4739 9285 54

H(15A) 8982 6636 9296 77

H(16A) 10300 6710 7990 68

H(17A) 9608 5214 6466 53

H(18) 7699 3168 6318 47

H(19A) 4577 191 6997 42

H(19B) 5639 1028 6258 42

H(20A) 8079 465 6569 51

H(21A) 7304 -2169 6270 93

H(21B) 5827 -1596 6160 93

H(21C) 6478 -2229 7282 93

H(22A) 9515 -855 7544 103

H(22B) 8552 -954 8482 103

H(23A) 10694 994 9118 165

H(23B) 9294 1674 8775 165

H(23C) 10398 1665 7953 165

H(25A) 3457 5111 3129 67

H(26A) 5357 4213 4166 76

H(27A) 7639 5779 4799 77

H(28) 8295 8066 4088 57

H(29A) 6125 8861 3362 78

H(31) 2085 9003 304 58

H(32A) 2215 8950 -1472 49

H(33) 4060 8017 -2103 70

H(34A) 5300 6881 -903 102

H(35) 5027 6616 950 65

H(37A) 1030 6693 3416 43

H(38) -959 4834 3435 48

H(39A) -1842 3238 1828 86

H(40A) -496 3507 426 49

H(41A) 1525 5348 495 48

H(42A) 2495 8779 3519 74

H(42B) 3778 9730 3020 74

H(43A) 671 9705 3313 62

H(44A) -1148 10667 2073 80

H(44B) -209 10671 1140 80

H(45A) -2306 8919 832 145

H(45B) -1638 8256 1934 145

H(45C) -834 8345 909 145

H(46A) 2731 11541 3655 121

H(46B) 1230 12062 3588 121

H(46C) 2042 12215 2567 121

_______________________________________________________________

**Table 6.** Torsion angles [deg] for sbi_0m.

________________________________________________________________

C(6)-C(1)-C(2)-C(3) 4(7)

C(1)-C(2)-C(3)-C(4) -6(6)

C(2)-C(3)-C(4)-C(5) 11(6)

C(3)-C(4)-C(5)-C(6) -12(6)

C(4)-C(5)-C(6)-C(1) 8(5)

C(4)-C(5)-C(6)-P(1) -176(3)

C(2)-C(1)-C(6)-C(5) -5(6)

C(2)-C(1)-C(6)-P(1) 179(3)

C(7)-P(1)-C(6)-C(5) -68(4)

C(19)-P(1)-C(6)-C(5) 52(4)

C(13)-P(1)-C(6)-C(5) 176(3)

C(7)-P(1)-C(6)-C(1) 108(4)

C(19)-P(1)-C(6)-C(1) -131(4)

C(13)-P(1)-C(6)-C(1) -8(4)

C(6)-P(1)-C(7)-C(12) 166(3)

C(19)-P(1)-C(7)-C(12) 49(3)

C(13)-P(1)-C(7)-C(12) -75(3)

C(6)-P(1)-C(7)-C(8) -19(4)

C(19)-P(1)-C(7)-C(8) -137(3)

C(13)-P(1)-C(7)-C(8) 99(3)

C(12)-C(7)-C(8)-C(9) -1(6)

P(1)-C(7)-C(8)-C(9) -175(3)

C(7)-C(8)-C(9)-C(10) 2(7)

C(8)-C(9)-C(10)-C(11) -3(6)

C(9)-C(10)-C(11)-C(12) 4(5)

C(8)-C(7)-C(12)-C(11) 2(4)

P(1)-C(7)-C(12)-C(11) 176(2)

C(10)-C(11)-C(12)-C(7) -3(4)

C(6)-P(1)-C(13)-C(14) 91(4)

C(7)-P(1)-C(13)-C(14) -25(3)

C(19)-P(1)-C(13)-C(14) -149(3)

C(6)-P(1)-C(13)-C(18) -83(3)

C(7)-P(1)-C(13)-C(18) 161(3)

C(19)-P(1)-C(13)-C(18) 38(3)

C(18)-C(13)-C(14)-C(15) 3(6)

P(1)-C(13)-C(14)-C(15) -170(3)

C(13)-C(14)-C(15)-C(16) -3(6)

C(14)-C(15)-C(16)-C(17) 4(7)

C(15)-C(16)-C(17)-C(18) -6(7)

C(16)-C(17)-C(18)-C(13) 5(5)

C(14)-C(13)-C(18)-C(17) -4(5)

P(1)-C(13)-C(18)-C(17) 170(2)

C(20)-O(1)-C(19)-P(1) -139(3)

C(6)-P(1)-C(19)-O(1) 177(2)

C(7)-P(1)-C(19)-O(1) -66(3)

C(13)-P(1)-C(19)-O(1) 55(3)

C(19)-O(1)-C(20)-C(22) 176(3)

C(19)-O(1)-C(20)-C(21) -73(4)

O(1)-C(20)-C(22)-C(23) -66(4)

C(21)-C(20)-C(22)-C(23) -177(3)

C(36)-P(2)-C(24)-C(25) 9(3)

C(42)-P(2)-C(24)-C(25) 124(3)

C(30)-P(2)-C(24)-C(25) -113(3)

C(36)-P(2)-C(24)-C(29) -167(3)

C(42)-P(2)-C(24)-C(29) -51(3)

C(30)-P(2)-C(24)-C(29) 71(3)

C(29)-C(24)-C(25)-C(26) -7(6)

P(2)-C(24)-C(25)-C(26) 177(3)

C(24)-C(25)-C(26)-C(27) 3(5)

C(25)-C(26)-C(27)-C(28) -6(5)

C(26)-C(27)-C(28)-C(29) 13(5)

C(27)-C(28)-C(29)-C(24) -16(6)

C(25)-C(24)-C(29)-C(28) 15(6)

P(2)-C(24)-C(29)-C(28) -169(4)

C(36)-P(2)-C(30)-C(35) -101(4)

C(24)-P(2)-C(30)-C(35) 24(4)

C(42)-P(2)-C(30)-C(35) 145(3)

C(36)-P(2)-C(30)-C(31) 73(4)

C(24)-P(2)-C(30)-C(31) -162(3)

C(42)-P(2)-C(30)-C(31) -42(4)

C(35)-C(30)-C(31)-C(32) 4(6)

P(2)-C(30)-C(31)-C(32) -170(3)

C(30)-C(31)-C(32)-C(33) -7(6)

C(31)-C(32)-C(33)-C(34) 6(6)

C(32)-C(33)-C(34)-C(35) -1(7)

C(31)-C(30)-C(35)-C(34) 1(6)

P(2)-C(30)-C(35)-C(34) 174(3)

C(33)-C(34)-C(35)-C(30) -2(8)

C(24)-P(2)-C(36)-C(37) 75(4)

C(42)-P(2)-C(36)-C(37) -43(4)

C(30)-P(2)-C(36)-C(37) -162(3)

C(24)-P(2)-C(36)-C(41) -96(3)

C(42)-P(2)-C(36)-C(41) 146(3)

C(30)-P(2)-C(36)-C(41) 27(4)

C(41)-C(36)-C(37)-C(38) 1(6)

P(2)-C(36)-C(37)-C(38) -170(3)

C(36)-C(37)-C(38)-C(39) -3(6)

C(37)-C(38)-C(39)-C(40) 3(5)

C(38)-C(39)-C(40)-C(41) -2(6)

C(39)-C(40)-C(41)-C(36) 1(6)

C(37)-C(36)-C(41)-C(40) 0(5)

P(2)-C(36)-C(41)-C(40) 172(3)

C(43)-O(2)-C(42)-P(2) 152(3)

C(36)-P(2)-C(42)-O(2) -67(3)

C(24)-P(2)-C(42)-O(2) 172(2)

C(30)-P(2)-C(42)-O(2) 50(3)

C(42)-O(2)-C(43)-C(46) 74(4)

C(42)-O(2)-C(43)-C(44) -158(3)

C(46)-C(43)-C(44)-C(45) -178(4)

O(2)-C(43)-C(44)-C(45) 59(4)

________________________________________________________________

Symmetry transformations used to generate equivalent atoms:

**Table 7.** Hydrogen bonds for sbi_0m [A and deg.].

____________________________________________________________________________

D-H...A d(D-H) d(H...A) d(D...A) <(DHA)
