# Supplementary material for: Facile synthesis of α-alkoxymethyltriphenylphosphonium iodides: new application of PPh3/I2
Source: Chem Cent J. 2018 May 17;12:62. doi: 10.1186/s13065-018-0421-6 (PMC5957017; doi:10.1186/s13065-018-0421-6)
Supplement: Supplementary file 5 — Additional file 5. Specimen NMR Spectra of alkoxymethyltriphenylphosphonium iodides. [file 13065_2018_421_MOESM5_ESM.docx]

**Additional file 5. Specimen NMR Spectra of alkoxymethyltriphenylphosphonium iodides**

| ^1^H NMR | ^31^P NMR |
| --- | --- |
|  |  |
|  |  |
|  |  |
