# Supplementary figures and images for: Facile synthesis of α-alkoxymethyltriphenylphosphonium iodides: new application of PPh3/I2
Source: Chem Cent J. 2018 May 17;12:62. doi: 10.1186/s13065-018-0421-6 (PMC5957017; doi:10.1186/s13065-018-0421-6)

**Additional file 6. Specimen NMR Spectrum of vinyl ether**

| 1H NMR | 13C NMR |
| --- | --- |
| 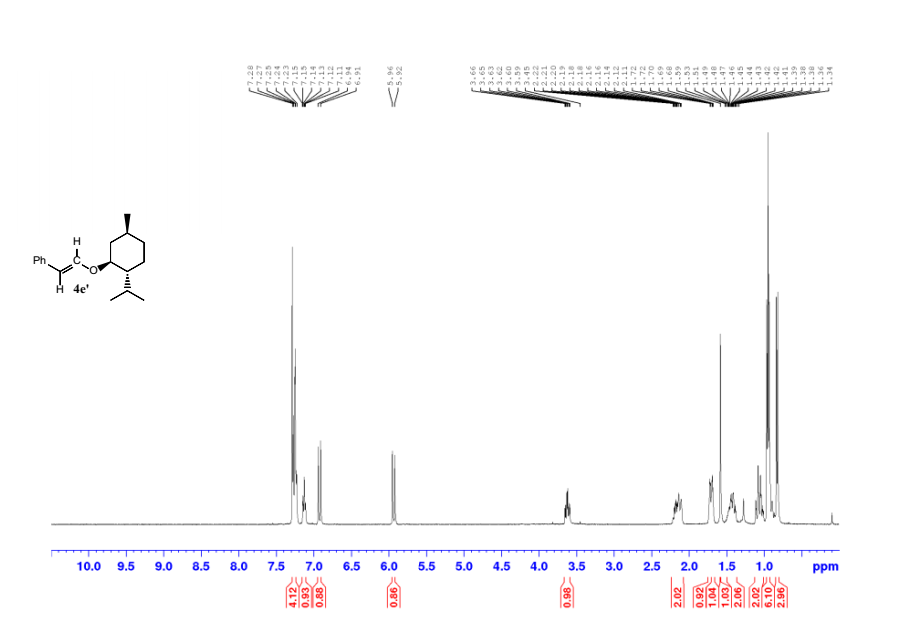 | 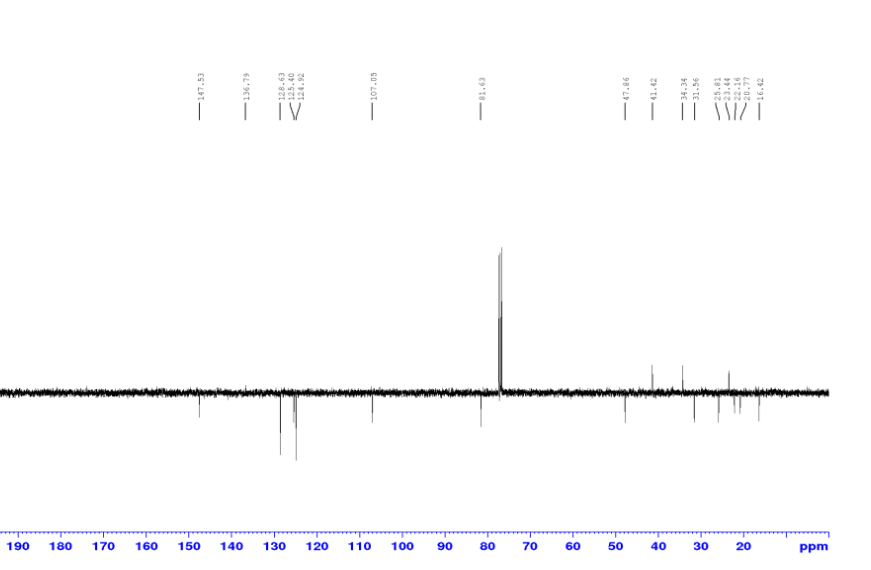 |

Supplement: Supplementary file 6 — Additional file 6. Specimen NMR Spectrum of vinyl ether. [file 13065_2018_421_MOESM6_ESM.docx]
